# Validity, reliability, and correlates of the Smartphone Addiction Scale–Short Version among Japanese adults

**DOI:** 10.1186/s40359-023-01095-5

**Published:** 2023-03-23

**Authors:** Toshitaka Hamamura, Nao Kobayashi, Taiki Oka, Issaku Kawashima, Yuki Sakai, Saori C. Tanaka, Masaru Honjo

**Affiliations:** 1 Healthcare Medical Group, Co-creation Division, KDDI research atlier, KDDI Research, Inc., 2 Chome-10-4 Toranomon, Mitano City, Tokyo, 105-0001 Japan; 2 National Center for Cognitive Behavior Therapy and Research, National Center of Neurology and Psychiatry, Tokyo, Japan; 3 Japan Society for the Promotion of Science, Tokyo, Japan; 4 Neuro Science Project AI Division, KDDI Research, Inc., Saitama, Japan; 5 Brain Information Communication Research Laboratory Group, Advanced Telecommunications Research Institutes International, Kyoto, Japan; 6 Department of Neuropsychiatry, Faculty of Life Sciences, Kumamoto University, Kumamoto, Japan; 7 Division of Information Science, Graduate School of Science and Technology, Nara Institute of Science and Technology, Nara, Japan

**Keywords:** SAS-SV, Problematic smartphone use, Smartphone addiction, Validity, Reliability, Japanese adults

## Abstract

**Background:**

The short version of the smartphone addiction scale (SAS-SV) is widely used to measure problematic smartphone use (PSU). This study examined the validity and reliability of the SAS-SV among Japanese adults, as well as cross-sectional and longitudinal associations with relevant mental health traits and problems.

**Methods:**

Datasets from a larger project on smartphone use and mental health were used to conduct two studies. Participants were adults aged over 20 years who carried a smartphone.

**Results:**

Study 1 (*n* = 99,156) showed the acceptable internal consistency and structural validity of the SAS-SV with a bifactor model with three factors. For the test-retest reliability of the SAS-SV, the intraclass correlation coefficient (ICC) was .70, 95% CI [.69, 70], when the SAS-SV was measured seven and twelve months apart (*n* = 20,389). Study 2 (*n* = 3419) revealed that when measured concurrently, the SAS-SV was strongly positively correlated with another measure of PSU and moderately correlated with smartphone use time, problematic internet use (PIU), depression, the attentional factor of impulsiveness, and symptoms related to attention-deficit hyperactivity disorder and obsessive-compulsive disorder. When measured 12 months apart, the SAS-SV was positively strongly associated with another measure of PSU and PIU and moderately associated with depression.

**Discussion:**

The structural validity of the SAS-SV appeared acceptable among Japanese adults with the bifactor model. The reliability of the SAS-SV was demonstrated in the subsequent seven- and twelve-month associations.

**Conclusion:**

The cross-sectional and longitudinal associations of the SAS-SV provided further evidence regarding PSU characteristics.

**Supplementary Information:**

The online version contains supplementary material available at 10.1186/s40359-023-01095-5.

## Background

Smartphones are electronic devices used by many individuals daily. For example, 74.3% of adults in Japan carried a smartphone as of 2021 [1]. In addition to internet access and instant communication with others, smartphones have brought many benefits, including assisting in medical and educational settings [2, 3]. Meanwhile, one meta-analytic study suggested that 23.3% of children and youth may experience problematic smartphone use (PSU) [4]. While most people use smartphones appropriately, some individuals suffer from the consequences of PSU.

Some scholars have proposed various conceptualizations of PSU. For example, Billieux and colleagues [5] suggested that PSU is multidimensional with several paths, such as excessive reassurance, impulsivity, and extraversion pathways, which lead to different problematic uses and behaviors. Another approach is conceptualizing PSU as a behavioral addiction. In addition to functional impairment, the characteristics of tolerance, withdrawal without use, and reckless use may be evident in PSU [6]. Meanwhile, some researchers have been cautious about conceptualizing PSU as an addiction and argued that problems originate from the content (e.g., gaming, social networking service, and video clips) rather than from the devices [7, 8]. To better understand PSU and the related problems, a recent review has suggested distinguishing between smartphone use and non-smartphone use since many individuals use different applications when using smartphone devices compared to non-smartphone devices (e.g., personal computers) [9]. Many applications, such as WhatsApp, Instagram, TikTok, and YouTube Shorts, are used mainly or only on smartphone devices.

The Smartphone Addiction Scale (SAS) [10] is one of the most widely used scales for assessing PSU [6]. The scale assesses the dimensions of PSU, including daily life disturbance, withdrawal, overuse, tolerance, positive anticipation, and cyberspace-oriented relationships. The short version of the SAS (SAS-SV) is also a widely used scale that has been translated into multiple languages [11]. While validity and reliability have been demonstrated mostly among adolescents and emerging adults, only a few studies have demonstrated the validity and reliability of the SAS-SV among adults in general, including Chinese, Belgian, and Moroccan adults [12–14]. Between 12.5 and 31.3% of individuals aged more than 18 years meet the cutoff score of the SAS-SV suggested in the original article [11, 12, 15–17].

Research over the last decade has revealed the association of PSU with other mental health problems. Meta-analytic studies have shown that PSU is positively moderately associated with impulsivity, depression and anxiety among emerging adults [18] and weakly associated with neuroticism [19]. Among cross-sectional studies, PSU, as measured by the SAS-SV, is strongly positively associated with depression among Saudi Arabian young adults, moderately associated among Chinese children and adolescents, and weakly associated among American university students [15, 20, 21]. PSU, as also measured by SAS-SV is also moderately positively associated with impulsiveness and self-esteem among US university students and weakly associated with social anxiety and sleep quality among Chinese adolescents [20–22]. Among those studies that have used the SAS, associations of PSU with attention-deficit hyperactivity disorder (ADHD) have been reported to be positive and strong among Korean adolescents [23]. Associations between PSU and  alcohol use disorder (AUD) have been reported to be negligible among Korean undergraduate students [24].

A few studies have investigated the longitudinal associations of PSU. PSU, as measured by the SAS-SV has been found to be moderately positively associated with depression and anxiety measured six and eighteen months later among Chinese college students [25]. Additionally, a medium six-month association between PSU, as measured by the Smartphone Addiction Inventory, and depression has been reported [26] among Chinese adolescents. Relatively few longitudinal studies have examined the long-term associations of PSU, and no studies are available among adults in general to the best of our knowledge.

In Japan, the validity of the SAS-SV has been shown among undergraduate students [17], and the results showed that the SAS-SV is associated with *hikikomori*, known as social withdrawal. However, the reliability and validity of the SAS-SV among adults, in general, is not well known. Hence, we pose the following two research questions. (1) is the SAS-SV a valid and reliable measure among Japanese adults? (2) How much is PSU, as measured by the SAS-SV, associated with other mental health traits and problems? Answering these questions can help researchers and clinicians assess PSU among Japanese adults and accumulate scientific evidence concerning its possible risks and consequences.

The purpose of this study was to examine the validity and reliability of the SAS-SV among Japanese adults and its associations with the relevant traits and outcomes of mental health. This study consists of two individual studies that used datasets for a larger project on mental health related to smartphone use. Parts of the data have been used in previously published studies [27, 28].

## Study 1

### Purpose

Study 1 examined the internal consistency and structural validity of the SAS-SV. This study conducted an item analysis for internal consistency, examined model fit for structural validity and investigated the test-retest reliability of the SAS-SV. Previous studies have demonstrated the test-retest reliability of PSU with a one-month interval [29], problematic internet use (PIU) with a one-month interval [30], and internet gaming disorder with two- and three-month intervals [31, 32]. We determined that seven- and twelve-month intervals may be justified given that the conditions of behavioral addictions, including internet gaming disorder and gambling disorder, are assessed over the past twelve months in the Diagnostic and Statistical Manual of Mental Disorders (5th ed., Text Revision; DSM-5TR) [33–35].

### Materials and methods

#### Participants and procedure

Participants were individuals registered in the sampling pool of a research marketing company. Inclusion criteria were (1) being aged 20 years or older and (2) living in the mid-western region of Japan (i.e., Kinki area). Participants received an online notification about the recruitment for participation in the study. After consenting to participate in this study, participants completed the provided questionnaires. Additional file 1: Table S1 shows participant sociodemographic characterstics.

**Fig. 1:**
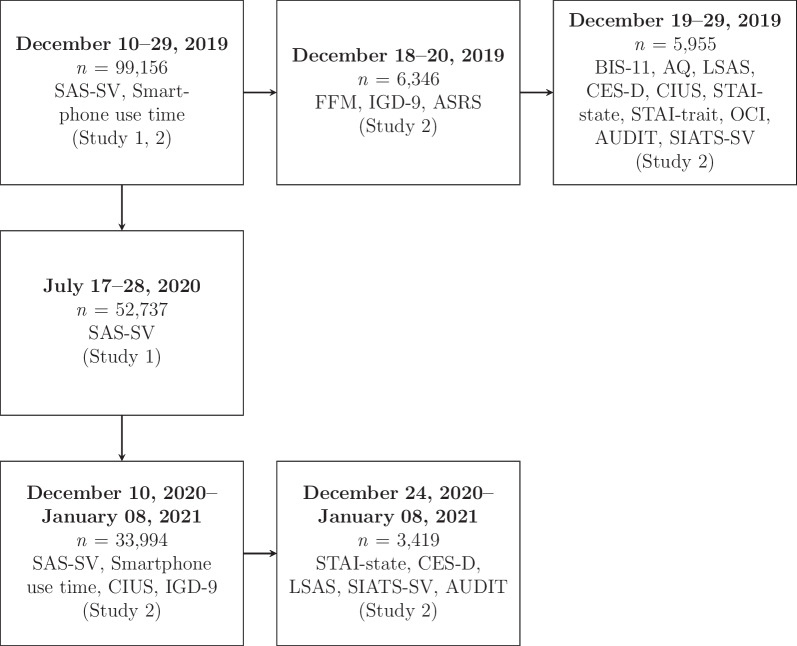
Flow chart of the study

The original project was extended to examine the associations of smartphone use with various mental health outcomes during the coronavirus disease 2019 (COVID-19) pandemic. This dataset from the extended project was used to examine the test-retest reliability. Participants answered a questionnaire at three-time points: December 2019, July 2020, and December 2020/January 2021 (see Fig. 1).

#### Measures

A Japanese version of the SAS-SV was used [11]. We used the version translated by the National Hospital Organization Kurihama Medical and Addiction Center [36]. Investigators received permission to use the scale in the current project. The scale comprises ten items on a six-point Likert scale (1 = strongly disagree, 6 = strongly agree). Item examples are “Feeling impatient and fretful when I am not holding my smartphone” and “Using my smartphone longer than I had intended.” The validated study suggested the unidimensional structure as a scale total [11].

#### Statistical analysis

Python 3.10.10 and R 4.1.0. were used for all statistical analyses [37, 38]. For item analyses, confirmatory factor analysis (CFA) was performed to calculate Cronbach’s alpha ($\alpha$) and McDonald’s omega ($\omega$) using the lavaan package (version 0.6.8) and the semTools package (version 0.5.5) [39, 40]. Cronbach’s $\alpha$ when an item was deleted, and item-total correlations were calculated using the psych package (version 2.1.9) [41]. Model fit was examined through CFA and considered acceptable if the comparative fit index (CFI) was above.90, the Tucker–Lewis index (TLI) was above.90, and the root mean square error of approximation (RMSEA) was below .08. When model fit was not adequate, a bifactor model was considered using omega hierarchical ($\omega _H$), omega hierarchical subscale ($\omega _{HS}$), explained common variance (ECV) and percentage of uncontaminated correlations (PUC) using the BifactorIndicesCalculator package (version 0.2.2) [42]. The scale was considered unidimensional when $\omega _H$ was above .80 and ECV above .50, and PUC above .70 [43].

To demonstrate the test-retest reliability of the SAS-SV across different time points, an intraclass correlation coefficient (ICC) was calculated using the irr package (version 0.84.1) [44]. Reliability was determined to be unacceptable, acceptable, good, and excellent when the correlation coefficient was below .70, between .70 and .79, between .80 and .89, and above .90, respectively [45]. Additionally, Spearman’s rank-ordered correlations ($\rho$) of paired time points were calculated.

### Results and discussion

#### Internal consistency and structural validity

In total, 99,156 participants provided responses, 45.70 % of which were women (*n* = 45,314). A total of 9034 participants (9%) were removed from the analysis because they reported not carrying any smartphones. Table 1 shows the item analysis of the SAS-SV. The corrected item-total correlations ranged between .52 and .77. When one item was removed from the scale, Cronbach’s $\alpha$ ranged between .49 and .71.

CFA showed that the model fit of the unidimensional structure of the SAS-SV was not acceptable, $\chi ^2$(35) = 59,677.03, *p* < .001, CFI = .86, TLI = .82, RMSEA = .14, Akaike information criterion (AIC) = 2,668,492, Bayesian information criterion (BIC) = 2,668,774. To examine an alternative factor structure, we followed the factor structure from the full scale (i.e., SAS) [10]. Items 1, 2 and 3 were endorsed as“Daily-life disturbance,” and Items 4, 5, 6, and 7 were endorsed as “Withdrawal.” Since Items 8, 9 and 10 belong to different factors, we grouped them as “Others.” Model fit improved with a bifactor model with the three-factor structure, $\chi ^2$(19) = 419.34, *p* < .001, CFI = .99, TLI = .97, RMSEA = .059, AIC = 174,920, BIC = 175,228, $\Delta$ $\chi$(16) = 40,298, *p* < .001. $\omega _H$, ECV and PUC were .76, .57 and .73, respectively. Table 2 shows the descriptive statistics of the SAS-SV with the bifactor model with the three-factor structure. While $\omega _H$ were just below .80, $\omega _{HS}$ were .40 or lower in all three subscale factors. Additional file 1: Fig. S1 shows the standardized factor loadings of the bifactor structure.

Cronbach’s $\alpha$ in this study (.88) was comparable to those .84 and .82 that have been reported among Chinese adults and Nigerian undergraduates [13, 46]. The item-total correlations in this study were higher, and the Cronbach’s $\alpha$ values when an item was deleted were lower compared to those previous studies. Regarding the factor structure, previous studies also reported a poor model fit of the one-factor structure among Chinese and Brazilian adolescents [20, 47]. In this study, the fit indices of the three-factor structure were above the acceptable level. Meanwhile, the reported $\omega _H$, $\omega _{HS}$, ECV and PUC values all support the unidimensionality of the bifactor model.

**Table 1:** Item analysis SAS-SV

Item	Description	Mean (SD)	Corrected item-total correlation	Cronbach’s $\alpha$ if item deleted
1	Missing planned work due to smartphone use	2.33 (1.28)	.71	.65
2	Having a hard time concentrating in class, while doing assignments, or while working due to smartphone use	2.15 (1.22)	.72	.66
3	Feeling pain in the wrists or at the back of the neck while using a smartphone	2.78 (1.50)	.52	.50
4	Won’t be able to stand not having a smartphone	2.97 (1.46)	.72	.68
5	Feeling impatient and fretful when I am not holding my smartphone	2.11 (1.16)	.76	.71
6	Having my smartphone in my mind even when I am not using it	2.09 (1.17)	.77	.71
7	I will never give up using my smartphone even when my daily life is already greatly affected by it.	2.66 (1.30)	.74	.70
8	Constantly checking my smartphone so as not to miss conversations between other people on Twitter or Facebook	2.12 (1.27)	.60	.57
9	Using my smartphone longer than I had intended	3.24 (1.46)	.67	.63
10	The people around me tell me that I use my smartphone too much.	2.26 (1.30)	.61	.58

**Table 2:** Descriptive statistics and intercorrelations of SAS-SV with a bifactor model

	Factor	Mean (SD)	Median	Range	$\omega _H$/$\omega _{HS}$	1	2	3	4
1	General	24.72 (9.40)	25	10–60	.76	–	.84	.91	.87
2	Daily-life disturbance	7.26 (3.25)	7	3–18	.40		–	.64	.62
3	Withdrawal	9.83 (4.26)	10	4–24	.37			–	.72
4	Others	7.62 (3.14)	8	3–18	.23				–

*SD*, Standard deviation. Spearman’s rank-ordered correlations ($\rho$) were calculated for the intercorrelations. All correlations were significant at *p* > .001

#### Test-retest reliability

A total of 33,994 participants provided responses at all three-time points, and 48.29% of participants were women. The agreement between different time points was fair, ICC = .70, 95% CI[.69, 70], *F*(33,993, 21,757) = 7.91, *p* < .001. The correlation coefficients of SAS-SV were $\rho$ = .70 between December 2019 and July 2020, $\rho$ = .69 between December 2019 and December 2020/January 2021, and $\rho$ = .73 between July 2020 and December 2020/January 2021 (*p* < .001).

The ICC was lower than the one-week reliability among Brazilian university students (ICC = .82), possibly due to the measurement intervals in this study. The correlation coefficients among the three time points in Study 2 were lower, but comparable to the one-week test-retest reliability of the SAS-SV ($\alpha$ = .76) among Chinese adults [13].

## Study 2

### Purpose

To evaluate the concurrent and predictive validity of the SAS-SV among Japanese adults, Study 2 also examined the cross-sectional and longitudinal associations of the SAS-SV with relevant traits and problems. Since this study was conducted using datasets established for another project, specific hypotheses were not formulated prior to data collection. Nevertheless, we expected that when measured concurrently, the SAS-SV would be strongly positively correlated with another measure of PSU; moderately correlated with smartphone use time, PIU, internet gaming disorder, impulsiveness, ADHD, and depression; and weakly correlated with neuroticism, anxiety, and AUD. For predictive validity, we expected that the SAS-SV would be associated with another measure of PSU, smartphone use time, PIU, internet gaming disorder, depression, and anxiety. For exploratory purposes, we examined the associations of the SAS-SV with other mental health problems, including obsessive-compulsive disorder (OCD) and autistic spectrum disorder (ASD).

### Materials and methods

#### Procedure and participants

Study 2 implemented a prospective study and used questionnaires administered in December 2019 (T1) and December 2020/January 2021 (T2) followed by additional questionnaires at each time point (see Fig. 1). Participants responded to the T1 additional questionnaires at two different time points to reduce the burden of study participation. Additionally, participants were recruited such that the distribution of the SAS-SV would be evenly divided by five in the additional questionnaires for an inclusion criterion of another study. Participants at T2 were those who completed the first questionnaire in July 2020, which was included in Study 1. All participants read and provided informed consent before answering each questionnaire.

#### Measures

*SAS-SV* The same version of the SAS-SV as that described in Study 1 was used.

*Another measure of PSU* The short version of the Smartphone-based Internet Addiction Tendency Scale (SIATS-SV) was used [48]. Of the 38 items from the original scale, [49], the short version comprises 16 items endorsed to a four-factor structure, namely, unsettled state of mind, regulation difficulty, smartphone incentives, and approval needs. The validity of both versions has been examined among Japanese high school students in the cited articles. The scale begins with the following statement: “This questionnaire asks about your smartphone use on a normal day.” Item examples are “I become restless when unable to use my smartphone” endorsed to the unsettled state mind factor; “I have failed to reduce my smartphone usage” endorsed to the regulation difficulty factor; “I always check social network services or emails first, even when I have other priorities” endorsed to the smartphone incentive factor; and “I feel lonely if others do not ‘like’ my posts” endorsed to the approval needs factor. To simplify the analysis, we used the scale total rather than the score for each factor. The Additional file 1 shows the acceptable structural validity of the bifactor model.

*Smartphone use time* Participants provided their self-reported average smartphone use time per day. Participants reported their typical smartphone use time on weekdays and weekends on a 12-point Likert scale (1 = less than one hour, 12 = more than 12 h). The total daily smartphone use time was calculated as (weekday day $\times$ 5 + weekend day $\times$ 2) / 7. Participants could also select another response, “I do not know,” which was treated as a missing response.

*PIU* The Compulsive Internet Use Scale (CIUS) was used [50, 51]. The scale consists of 14 items answered on a five-point Likert scale (1 = never, 5 = always). Examples of items include “How often do you find it difficult to stop using the internet when you are online?” and “How often do you think about the internet, even when not online?” This study used a one-factor structure, as demonstrated in the cited articles.

*Internet gaming disorder* Lemmen’s short version of the Internet Gaming Disorder Scale (IGD-9) was used [35, 52]. On a binary response format, this unidimensional scale consists of nine items that ask for the criterion of internet gaming disorder in the DSM-5 [33]. Items include “Have there been periods when all you could think of was the moment that you could play a game?” and “Have you felt unsatisfied because you wanted to play more?”

*AUD* The Alcohol Use Disorder Identification Test (AUDIT) was used [53, 54]. This unidimensional scale consists of ten items answered on a five-point Likert scale, and the response options differ for each item. The AUDIT measures the levels of AUD by assessing drinking quantity and frequency, dependence symptoms, and harmful alcohol use. Items include “How often during the last year have you found that you were not able to stop drinking once you had started?” and “Have you or someone else been injured as a result of your drinking?”

*ADHD* The Adult ADHD Self-Report Scale-V.1.1 Symptoms Checklist (ASRS-V1.1) was used [55, 56]. Using a five-point Likert scale (0 = rarely, 4 = very often), the scale consists of a six-item screening portion (Part A) and a 12-item additional portion (Part B). Item examples are “How often do you have trouble wrapping up the final details of a project, once the challenging parts have been done?” in Part A and “How often do you make careless mistakes when you have to work on a boring or difficult project?” in Part B. The scale total was used in this study.

*ASD* The Autism-Spectrum Quotient (AQ) was used [57, 58]. This unidimensional scale consists of 50 items answered on a four-point Likert scale (0 = definitely agree, 3 = definitely disagree). Item examples are “I prefer to do things with others rather than on my own” and “I prefer to do things the same way over and over again.”

*Impulsiveness* The Barratt Impulsiveness Scale, Version 11 (BIS-11) was used [59, 60]. The scale consists of 30 items answered on a four-point Likert scale (1 = rarely/never, 4 = almost always/always). Items include “I plan tasks carefully,” “I do things without thinking,” and “I make-up my mind quickly.” As recommended by the authors, second-order factors, namely, attentional, motor, and nonplanning, were used.

*Big-five personality traits* The five-factor model (FFM) was used to measure openness, conscientiousness, extraversion, agreeableness, and neuroticism [61, 62]. This scale with a higher-order structure consists of 60 items answered on a seven-point Likert scale (1 = not applicable at all, 7 = very applicable). Items include “I am flexible” in terms of openness, “I like order” in terms of conscientiousness”, “I am sociable” in terms of extroversion, “I sympathize with other people” in terms of agreeableness, and “I often feel blue” in terms of neuroticism.

*OCD* The Obsessive-Compulsive Inventory (OCI) was used [63, 64]. Answered on a five-point Likert scale, the OCI consists of 42 items consisting of seven subscales: washing, checking, doubting, ordering, obsessing, hording, and mental neutralizing. Item examples are “Unpleasant thoughts come into my mind against my will and I cannot get rid of them,” and “I have to review mentally past events, conversations and actions to make sure that I didn’t do something wrong.” This study used the scale total.

*Depression* The Center for Epidemiological Studies Depression (CES-D) was used [65, 66]. This unidimensional scale consists of 20 items scored on a four-point scale (0 = rarely or none of the time [less than 1 day] to 3 = most or all of the time [5–7 days]). Example items are “I was bothered by things that usually don’t bother me” and “I did not feel like eating: my appetite was poor.”

*Social anxiety* The Liebowitz Social Anxiety Scale (LSAS) was used [67, 68]. On a four-point Likert scale (0 = never to 3 = usually), the LSAS consists of 24 items that measure fear/anxiety and 24 items that measure avoidance related to social interaction and performance. Items include “Telephoning in public” and “Participating in small groups.” We used the scale total by summing all 48 items.

*State and trait anxiety* The State-Trait Anxiety Inventory (STAI) was used [69, 70]. The scale consists of 20 items that measure trait anxiety and 20 items that measure state anxiety, which are answered on a four-point Likert scale. Items include “I am worried” and “I feel nervous” for state anxiety and “I lack self-confidence” and “I am a steady person” for trait anxiety.

We measured SAS-SV, SIATS-SV, smartphone use time, CIUS, IGD-9, AUDIT, CES-D, LSAS, and STAI-state both at T1 and T2, while ASRS, AUDIT, BIS-11, FFM, OCI, and STAI-trait were measured only at T1.

#### Statistical analysis

Python 3.10.10 and R 4.1.0 were used for all statistical analyses [37, 38]. To examine the associations between paired variables, Spearman’s rank correlation coefficients ($\rho$) were calculated. We calculated McDonald’s $\omega$ to show the internal consistency of each measure using the lavaan package (version 0.6.8) and semTools (version 0.5.5) [39, 40]. Multiple imputations using the mice package (version 3.14.0) [71] were used to subset missing items. Participants with inconsistent responses (e.g., answering 1 on all items despite the presence of reverse-scored items) were removed from the analysis. Effect sizes were regarded as high/strong, medium/moderate, and low/weak when the correlation coefficients were above .50, between .49 and .30, and between .29 and .10, respectively, according to the convention used for Pearson’s product-moment correlation coefficient [72].

### Results and discussion

A total of 3419 participants completed all the questionnaires up to T2. There were missing responses from 76 participants (1.28%). A total of 269 participants (4.52%) were removed from the analysis because of their inconsistent responses. These participants 1) provided the minimum or maximum value of the response scale despite the reverse items on FFM, AQ, CES-D, or STAI-trait; 2) reported drinking three standard drinks on the second item of AUDIT while reporting never drinking this quantity on the third item of AUDIT; or 3) reported the identical time between their sleep wake-up times, a measure not used in this study. Table 3 shows the mean, standard deviation, median, range, and McDonald’s $\omega$ of the measured variables, as well as the correlation coefficient with the SAS-SV at T1.

For concurrent validity, correlation coefficients at T1 were calculated. The SAS-SV was strongly positively correlated with the SIATS-SV (another measure of PSU). The SAS-SV was moderately positively associated with CIUS (PIU), smartphone use time, ASRS (ADHD), CES-D (depression), OCI (OCD), and the attentional factor of BIS-11 (impulsiveness). Moreover, the SAS-SV was weakly positively correlated with IGD-9 (internet gaming disorder), AUDIT (AUD), STAI-trait (trait anxiety), STAI-state (state anxiety), LSAS (social anxiety), FFM neuroticism, and the nonplanning and motor factors of BIS-11 (impulsiveness). Furthermore, the SAS-SV was weakly negatively associated with FFM conscientiousness and agreeableness. These cross-sectional associations demonstrated the concurrent validity of the SAS-SV among Japanese adults.

For predictive validity, the SAS-SV at T1 was strongly positively correlated with the SIATS-SV (another measure of PSU) and CIUS (PIU) at T2. Positive moderate associations were found with smartphone use time and CES-D (depression) at T2. Finally, the SAS-SV at T1 was weakly positively correlated with IGD-9 (internet gaming disorder), STAI-state (state anxiety), and LSAS (social anxiety) at T2.

**Table 3:** Descriptive statistics and correlation coefficient with SAS-SV at T1

Measure	Time	Mean (SD)	Median	Range	$\omega$	$\rho$	*p*-value
SAS-SV	T1	25.75 (10.04)	26	10–60	.92	–	–
	T2	24.30 (9.65)	25	10–60	.92	.72	<.001
SIATS-SV	T1	27.65 (10.80)	25	16–78	.87	.62	<.001
	T2	29.77 (11.33)	28	16–76	.87	.59	.59
Smartphone use time	T1	2.49 (1.96)	2	0.5–12.5	–	.47	<.001
	T2	2.47 (1.85)	2	0.5–12.5	–	.41	.41
CIUS	T1	12.94 (10.25)	12	0–56	.93	.49	<.001
	T2	15.23 (10.37)	15	0–56	.94	.60	.60
IGD-9	T1	0.56 (1.35)	0	0–9	.81	.23	<.001
	T2	0.68 (1.59)	0	0–9	.85	.26	<.001
AUDIT	T1	5.21 (5.57)	3	1–36	.85	.11	<.001
	T2	4.33 (5.64)	2	0–39	.85	.08	<.001
CES-D	T1	16.32 (10.65)	14	0–60	.90	.30	<.001
	T2	15.29 (10.48)	13	0–60	.90	.30	<.001
LSAS	T1	49.05 (29.92)	46	0–144	.97	.20	<.001
	T2	54.36 (32.77)	52	0–144	.97	.13	<.001
STAI-state	T1	45.43 (11.33)	46	20–80	.92	.25	<.001
	T2	49.96 (10.84)	50	20–80	.92	.22	<.001
STAI-trait	T1	47.00 (11.34)	48	20–80	.92	.26	<.001
OCI	T1	30.98 (29.42)	20	0–154	.98	.37	<.001
ASRS	T1	18.60 (10.29)	18	0–68	.92	.37	<.001
AQ	T1	22.37 (6.93)	23	1–46	.73	.16	<.001
BIS-11: Attentional	T1	17.36 (3.44)	17	10–31	.65	.30	<.001
BIS-11: Motor	T1	22.15 (4.59)	22	12–45	.71	.13	<.001
BIS-11: Nonplanning	T1	34.03 (4.80)	34	20–52	.75	.25	<.001
FFM: Openness	T1	46.47 (11.21)	47	12–84	.91	.01	.68
FFM: Conscientiousness	T1	52.58 (10.37)	52	12–84	.89	–.16	<.001
FFM: Extroversion	T1	49.18 (13.02)	49	12–84	.93	–.05	.003
FFM: Agreeableness	T1	52.47 (10.36)	52	12–84	.89	–.16	<.001
FFM: Neuroticism	T1	51.74 (14.75)	52	12–84	.95	.26	<.001

*AQ*, Autism-Spectrum Quotient; *ASRS*, Adult ADHD Self-Report Scale; *AUDIT*, Alcohol Use Disorder Identification Test; *BIS-11*, Barratt Impulsiveness Scale, Version 11.; *CES-D*, Center for Epidemiological Studies Depression; *CIUS*, Compulsive Internet Use Scale; *IGD-9*, *FFM,* The five-factor model; Internet Gaming Disorder Scale; *LSAS*, Liebowitz Social Anxiety Scale; *OCI*, Obsessive-Compulsive Inventory; *SAS-SV*, Smartphone Addiction Scale–Short Version; *SIATS-SV*, Short Version of the Smartphone-based Internet Addiction Tendency Scale; *STAI*, The State-Trait Anxiety Inventory, *T1*, December 2019; *T2*, December 2020/January 2021

## General discussion

This study examined the validity and reliability of the SAS-SV among Japanese adults in general. By testing the validity of the SAS-SV, this study also showed the cross-sectional and longitudinal associations of PSU with relevant mental health traits and problems. Study 1 revealed the acceptable internal consistency of the SAS-SV. Model fit was not adequate with the unidimensional model; however, in the bifactor model it appeared acceptable. As the multidimensional structure of the SAS-SV was suggested in a previous study among Chinese adolescents [20], future studies may take this alternative factor structure into account, and the scale total may be used.

Study 1 also revealed the acceptable test-retest reliability of the SAS-SV over periods of seven and twelve months. The correlation coefficients among the three time points were strong; this finding suggests that conditions of PSU conceptualized as addiction can persist for at least twelve months and that the SAS-SV can reliably measure PSU among Japanese adults. While test-retest reliability has been reported generally within one month, the seven- and twelve-month associations in this study offer unique insight into the reliability of the SAS-SV and constructs related to PSU.

Study 2 demonstrated the concurrent and predictive validity of the SAS-SV and its associations with mental health traits and problems. The strong and moderate association between the SAS-SV and CIUS suggests overlapping characteristics between PSU and PIU, while the weak association between the SAS-SV and IGD-9 suggests characteristics of PSU that are distinctive from those of internet gaming disorder.

Notably, the SAS-SV was moderately positively associated with depression and weakly associated with state anxiety and social anxiety both concurrently and twelve months later. The medium-sized longitudinal association between the SAS-SV and depression was comparable to the moderate association found among Chinese adolescents, and undergraduate students [24, 26]. Although this study did not recruit participants with higher levels of PSU, these associations have strengthened the evidence that internalizing problems are comorbid symptoms of PSU.

The cross-sectional associations of the SAS-SV with impulsivity and ADHD found in this study confirm the associations found in previous studies [18, 22, 23]. This study suggests that medium-sized associations with ADHD and the inattention aspect of impulsiveness stand out as particularly relevant with regard to PSU. Individuals with higher tendencies toward inattention may exhibit more frequent behaviors of checking smartphones, which are often readily accessible throughout the day. We also found a positive but small association of PSU with ASD, while this evidence of an association is scarce in the literature. Possibly, some individuals on the higher end of the ASD spectrum may find structured interactions on smartphones more attractive than real-world interactions, thereby resulting in PSU for such individuals.

The negative and weak cross-sectional association with conscientiousness and a positive, weak association with neuroticism found in this study were consistent with the findings of meta-analytic studies among adolescents [18, 19]. The small-sized association between PSU and agreeableness were a surprising result. One possible explanation for this finding is that individuals who identify themselves as being less warm and friendly toward others may prefer online interactions in which social harmony is not expected as much as it is in offline interactions. However, this association with agreeableness should be confirmed through future studies.

The medium-sized cross-sectional association between PSU and OCD found in this study may be explained by the overlapping characteristics related to loss of control in PSU. Only one empirical study has reported a positive association between PSU and OCD among adults [73], and thus, further studies are necessary to establish this association. The associations between the SAS-SV and AUDIT were negligible both concurrently and twelve months later. A stronger association has been reported among US undergraduates [22]. However, the finding in this study is consistent with those of a few other studies claiming an unrelated association between PSU and AUD, such as those conducted among Korean adolescents and Swiss vocational students [23, 74].

### Limitations

There are limitations in this study. First, this study used convenience sampling by recruiting participants registered at a research marketing company. Thus, the sample in this study may not accurately represent the Japanese adult population in general. Second, all questionnaires were self-reported, and answering them required a considerable amount of time and effort. Thus, participants’ motivations and interpretations of the items may have resulted in biased responses. Third, the questionnaires were administered during the COVID-19 pandemic in Japan. Factors such as temporarily spending more time at home and not meeting with other people in person may have affected the participants’ responses, including those regarding their smartphone use.

## Conclusions

This study showed acceptable psychometric properties of the SAS-SV using the bifactor model with a three-factor structure among Japanese adults. While relatively more studies on this topic have been conducted among children and youth, PSU is also a concern for adults as it affects their mental health and daily activities, including social, financial, and occupational aspects. Future studies may examine whether the SAS-SV may be a valid and reliable scale to use among those who seek treatment related to PSU. Additionally, this study showed the association of PSU, as measured by the SAS-SV, with psychopathological traits such as impulsiveness and neuroticism and psychopathological measures such as ADHD, internet gaming disorder, OCD, depression, and anxiety. These associations suggest that PSU is a clinically relevant construct; thus, future studies may take PSU into account when studying mental health, as human-computer interactions have become essential in many lives.

## Supplementary Information

**Additional file 1**: This file shows sociodemographic characteristics of the participants and structural validity of SAS-SV and SIATS-SV

## Data Availability

The datasets generated and/or analyzed during the current study are not publicly available due to the policy of the institution that funded this study but may be available from the corresponding author on reasonable request.

## Abbreviations

AIC: Akaike information criterion.
AQ: Autism-Spectrum Quotient.
ASRS: Adult ADHD Self-Report Scale.
AUDIT: Alcohol Use Disorder Identification Test.
BIC: Bayesian information criterion.
BIS-11: Barratt Impulsiveness Scale, Version 11.
CES-D: Center for Epidemiological Studies Depression.
CFI: Comparative fit index.
CIUS: Compulsive Internet Use Scale.
DSM: Diagnostic and Statistical Manual of Mental Disorders.
ECV: Explained common variance.
FFM: The five-factor model.
IGD-9: Internet Gaming Disorder Scale.
LSAS: Liebowitz Social Anxiety Scale.
OCI: Obsessive-Compulsive Inventory.
PIU: Problematic internet use.
PSU: Problematic smartphone use.
PUC: Percentage of uncontaminated correlations.
RMSEA: Root mean square error of approximation.
SAS: Smartphone Addiction Scale.
SAS-SV: Smartphone Addiction Scale–Short Version.
SIATS-SV: Short version of the Smartphone-based Internet Addiction Tendency Scale.
STAI: The State-Trait Anxiety Inventory.
TLI: Tucker–Lewis index.
T1: December 2019.
T2: December 2020/January 2021.
