# Supplementary material for: Validity, reliability, and correlates of the Smartphone Addiction Scale–Short Version among Japanese adults
Source: BMC Psychol. 2023 Mar 23;11:78. doi: 10.1186/s40359-023-01095-5 (PMC10034913; doi:10.1186/s40359-023-01095-5)
Supplement: Supplementary file 1 — Additional file 1: This file shows sociodemographic characteristics of the participants and structural validity of SAS-SV and SIATS-SV [file 40359_2023_1095_MOESM1_ESM.pdf]

Supplementary material

Table S1: Sociodemographic Characteristics of the Participants

Characteristics	n	%
Sex		
Men	26,498	50.25%
Women	26,239	49.75%
Employment		
Public official	939	3.92%
Manager/board member	538	2.24%
Company worker (administrative)	3,746	15.63%
Company worker (technical)	2,567	10.71%
Company worker (other)	4,229	17.64%
Self-employed	1,321	5.51%
Freelance	485	2.02%
Homemaker	3,454	14.41%
Part time	3,951	16.48%
Student	428	1.79%
Other	749	3.12%
Unemployed	1,565	6.53%

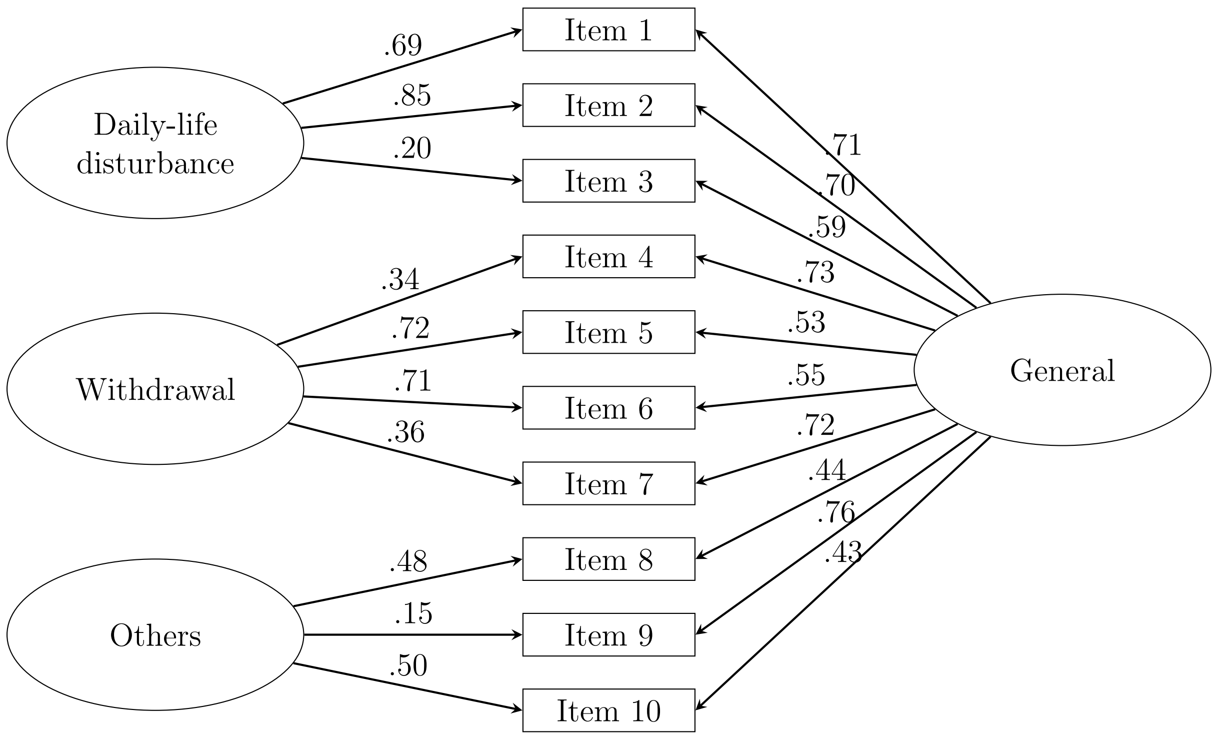

Figure S1. A path diagram of the bifactor model of the Japanese version of the Smartphone Addiction Scale–Short Version (SAS-SV) with standardized factor loadings.

Structural validity of SIATS-SV

Previously, the short version of the Smartphone-based Internet Addiction Tendency Scale (SIATS-SV) has been validated as a higher-order model. To use the scale total in this study, the structural validity of the bifactor model was examined in Study 2 with 5,686 participants after removing inconsistent responses. The results showed that the fit indices of the bifactor model were acceptable and better than those of the higher-order model (see Table S2). ω_H, Table S3 shows the intercorrelations and descriptive statistics of the bifactor model. ECV and PUC were .84, .64 and .80, respectively. Figure S2 shows the standardized factor loadings of the bifactor structure. Low factor loadings on Item 1 and Item 4 suggest a reconsideration of item selection for future studies.

Table S2: Model comparison of SIATS-SV

Model	CFI	TLI	RMSEA	AIC	BIC	*df*	χ^2	*p*-value
Higher-order model	.95	.94	.53	203,658	204,043	78	1,390.40	$< .001$
Bi-factor model	.98	.96	.04	204,889	205,128	100	2,665.87	$< .001$
Difference (Δ)						22	867	$< .001$

Note. AIC = Akaike information criterion. BIC = Bayesian information criterion. CFI = Comparative fit index. RMSEA = Root mean square error of approximation. TLI = Tucker-Lewis index.

Table S3: Intercorrelations and descriptive statistics of SIATS-SV with a bifactor model

	Factor/Statistics	1	2	3	4	5
1	General	–	.80	.88	.91	.65
2	Unsettled state of mind		–	.66	.67	.45
3	Regulation difficulty			–	.78	.41
4	Smartphone incentives				–	.45
5	Approval needs					–
	Mean	27.65	6.10	7.48	7.82	6.25
	SD	10.80	2.81	3.72	3.41	3.01
	Median	25	5	6	7	5
	Range	16–78	4–20	4–20	4–20	4–20
	ω_H/ω_{HS}	.84	.07	.43	.32	49

Note. SD = Standard deviation. Spearman's rank-ordered correlations (ρ) were calculated for the intercorrelations. All correlations were significant at $p > .001$

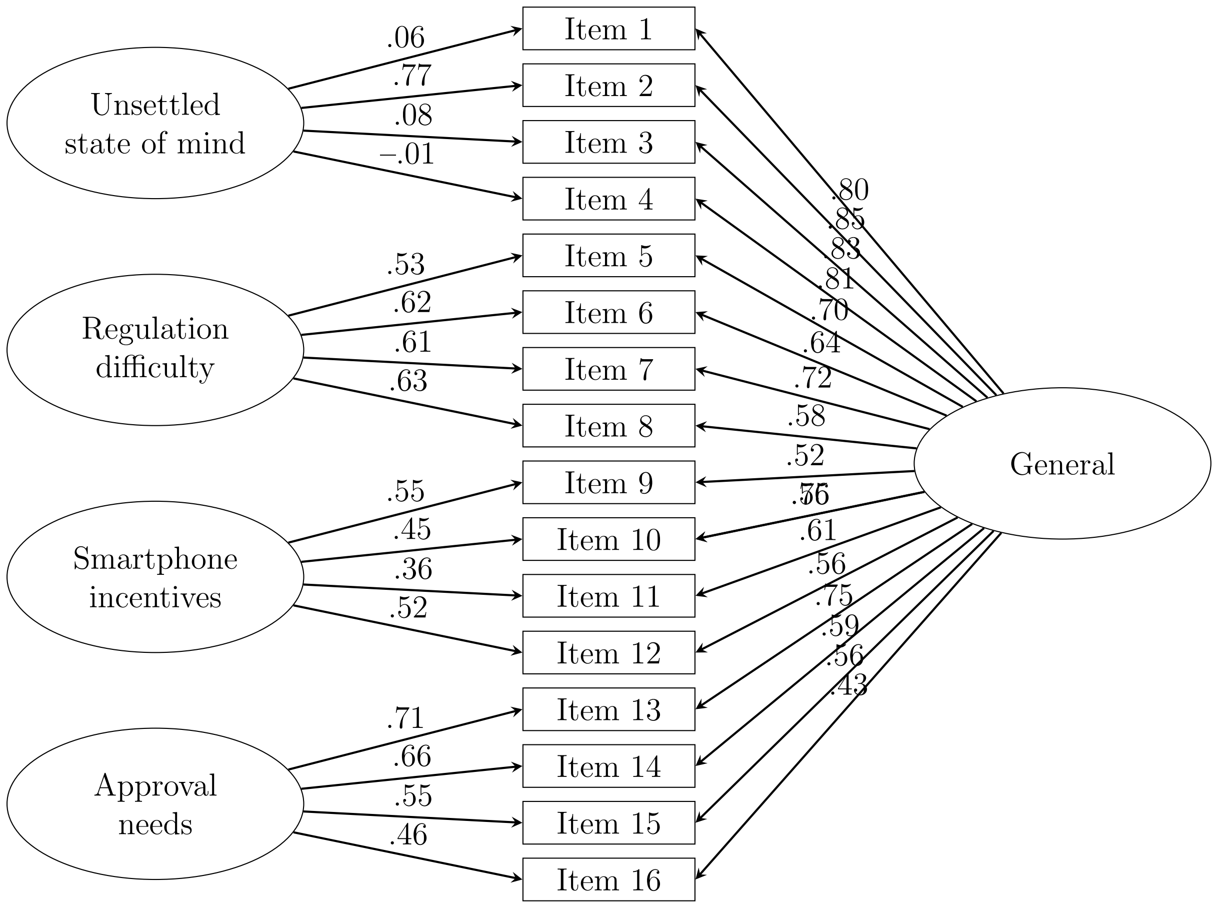

Figure S2. A path diagram of the bifactor model of the short version of the Smartphone-based Internet Addiction Tendency Scale (SIATS-SV) with standardized factor loadings.
